# Practical issues encountered while determining Minimal Clinically Important Difference in Patient-Reported Outcomes

**DOI:** 10.1186/s12955-020-01398-w

**Published:** 2020-05-27

**Authors:** Pascal Woaye-Hune, Jean-Benoit Hardouin, Paul-Antoine Lehur, Guillaume Meurette, Antoine Vanier

**Affiliations:** 1 Inserm, Université Bretagne-Loire - Université de Nantes - Université de Tours, UMR U1246 SPHERE “Methods in patient-centered outcomes and health research”, Nantes, France; 2 Internal Medicine Department, University Hospital of Nantes, Nantes, France; 3 Unit of Methodology and Biostatistics, University Hospital of Nantes, Nantes, France; 4 Digestive Surgery Department, University Hospital of Nantes, Nantes, France

**Keywords:** Minimal clinically important difference, Minimal important difference, Patient-reported outcomes, Methodology, Missing data, Longitudinal modeling

## Abstract

**Background:**

Using a real dataset, we highlighted several major methodological issues raised by the estimation of the Minimal Clinically Important Difference (MCID) of a Patient-Reported Outcomes instrument. We especially considered the management of missing data and the use of more than two times of measurement. While inappropriate missing data management and inappropriate use of multiple time points can lead to loss of precision and/or bias in MCID estimation, these issues are almost never dealt with and require cautious considerations in the context of MCID estimation.

**Methods:**

We used the LIGALONGO study (French Randomized Controlled Trial). We estimated MCID on the SF-36 General Health score by comparing many methods (distribution or anchor-based). Different techniques for imputation of missing data were performed (simple and multiple imputations). We also consider all measurement occasions by longitudinal modeling, and the dependence of the score difference on baseline.

**Results:**

Three hundred ninety-three patients were studied. With distribution-based methods, a great variability in MCID was observed (from 3 to 26 points for improvement). Only 0.2 SD and 1/3 SD distribution methods gave MCID values consistent with anchor-based methods (from 4 to 7 points for improvement). The choice of missing data imputation technique clearly had an impact on MCID estimates. Simple imputation by mean score seemed to lead to out-of-range estimate, but as missing not at random mechanism can be hypothesized, even multiple imputations techniques can have led to an slight underestimation of MCID. Using 3 measurement occasions for improvement led to an increase in precision but lowered estimates.

**Conclusion:**

This practical example illustrates the substantial impact of some methodological issues that are usually never dealt with for MCID estimation. Simulation studies are needed to investigate those issues.

**Trial registration:**

NCT01240772 (ClinicalTrials.gov) registered on November 15, 2010.

## Background

Nowadays, medical practice tends to be patient-centered, after being mostly based on objective outcomes. Subjective concepts like quality of life or satisfaction are now as relevant endpoints as mortality in clinical studies. Since they can be assessed mostly by patients’ speech, these concepts are at best reported directly by the patient himself without interpretation by a clinician with instruments called Patient-Reported Outcomes (PRO) [1]. These instruments are increasingly used in studies and clinical practice, since it gives the patient a central place in his medical care.

However, one of the major issues with the use of PRO is to correctly identify whether a change in scores is relevant or not, beyond statistical significance. Indeed, statistical significance only tells an observed difference is unlikely to have occurred by sampling hazard alone, but it does not tell if this difference has meaning, especially for the patient. For instance, an improvement of four points on a Health-Related Quality of Life (HRQoL) score after an intervention can be enough to reach statistical significance with appropriate sample size, but it can be hard to tell anyway if it is a meaningful difference.

To this end, in 1989 Jaeshcke defined the concept of the Minimal Clinically Important Difference (MCID), as “*the smallest difference in score in the domain of interest which patients perceive as beneficial and which would mandate, in the absence of troublesome side-effects and excessive cost, a change in the patient’s management*” [2]. Thus, determining the MCID of a PRO is interesting in clinical practice and research. For instance, a clinician wishing to evaluate his patients before and after a treatment can interpret the score difference by comparing it to the MCID. In clinical research, the MCID allows to calculate the number needed to treat in a clinical trial where the effect of a treatment is evaluated with a PRO.

Several methods have been proposed for MCID determination, classified as distribution-based and anchor-based methods mainly [3, 4]. It will be more detailed below, but briefly, distribution-based methods use the variability of overall responses to estimate MCID. They don’t consider the patient’s perspective, but still are useful because easy to estimate. The anchor-based methods use an external indicator (the anchor) to classify patients as improved or worsened and link it to the score difference. They can consider the patient’s point of view and are often assumed as the best way to estimate MCID [5].

Nonetheless, major methodological issues still need to be explored about MCID determination. The MIDIPRES project (Minimal clinically Important DIfference determination for patient reported outcomes in Presence of REsponse Shift), aims at defining the most adequate method for MCID determination. Among others, relevant existing methods will be compared using simulation studies. In these studies, responses to PRO will be generated by models. To adequately simulate data, parameters to account for are numerous. Certain categories of issues are frequently discussed in reviews about MCID estimation [4, 6, 7]. Nonetheless, as shown in a systematic review of all the methods used for MCID determination on empirical data since the inception of the concept, some of these issues are massively overlooked, or at least dealt with in an inappropriate manner [8]. Therefore, their impact on empirical data is still not systematically investigated. First, there must be at least a moderate correlation between the anchor and the measured score, to allow linking one with the other [6]. Second, very few studies consider missing values. There are often numerous in PRO data, but almost all the studies on MCID estimation on empirical data assume a complete case analysis is the appropriate data analysis. Nonetheless, depending on the missingness mechanism, these missing data cannot be ignored, since their exclusion can lead to a loss in precision and/or bias in estimation due to the non-representativeness of the complete-case sample. The appropriate method to deal with these missing values in the context of MCID estimation should be approached with great caution. Third, most of the time, patients are assessed on only two times of measurement. The few studies that consider more than two times of measurement generally don’t take the correlation of repeated measures into account and simply estimate an overall mean pooling data of each time of measurement, which is not appropriate [8]. Finally, it is admitted MCID estimate could be influenced by the baseline score level, but it is an issue that is still mostly overlooked [7–10].

To help planning adequate simulation studies, we wanted to investigate the potential impact of some of the aforementioned issues by comparing numerous methods of MCID determination to estimate the variability of MCID value, using a real dataset (the LIGALONGO study [11], a randomized control trial measuring HRQoL as secondary endpoint) as an illustration. Particularly, we aimed to investigate the methodological challenges of dealing with missing data in the context of MCID estimation and the variability of MCID estimates when managing missing data with recommended techniques. Another point of consideration was to investigate the impact of incorporating more than two times of measurement on MCID estimation. A last issue was to investigate the need in this context to consider baseline value in HRQL score in MCID estimation.

## Methods and patients

### Determination methods of MCID

#### Distribution-based methods

Distribution-based methods use the variability of overall responses to estimate MCID. They did not incorporate any form of external assessment about clinical meaning or patients’ perspective. Thus, some authors consider distribution-based MCID estimates only lead to what is called Smallest Detectable Differences (SDD): a statistical definition for quantifying the significance of differences [12, 13]. Nonetheless, distribution-based MCID estimates are easy to compute. In addition, as there is today no consensus of the best method to estimate MCID, some authors recommend what is called “triangulation” which corresponds to assess MCID using multiple methods of estimation to provide au plausible range where the true MCID could be [14]. In this context, distribution-based methods are still often considered as adding info about MCID estimation. We can distinguish two approaches of distribution-based methods.

1. *Those only based on the score change*

*Effect-Size (ES)* is obtained by dividing the difference in mean scores from baseline $\overline{x_1}$ to post-intervention $\overline{x_2}$ by the standard-deviation of the baseline score (SD_b_): $\frac{\overline{x_2}-\overline{x_1}}{SD_b\ }$ [15]. Cohen [16] empirically defined an effect size of 0.2 as small, 0.5 as moderate, and 0.8 as large. Then, one can consider 0.2 SD_b_, 1/3 SD_b_ or 0.5 SD_b_ to determine the MCID (equated to a minimal standardized observed change).

*Standardized response mean (SRM)* is defined by dividing the difference in mean scores from baseline to post-intervention by the SD of that difference (SD_ch_) [17, 18], or $\frac{\overline{x_2}-\overline{x_1}}{SD_{ch}}$. MCID is then considered as 0.2 SD_ch_, 1/3 SD_ch_ or 0.5 SD_ch_. SD_ch_ tends to diminish with increasing sample size. Thus, the SRM becomes increasingly dependent of the sample sizes for a constant SRM [13].

2. *Those based on the instrument capacity to detect a change beyond measurement error*

*Standard Error of Measurement (SEM),* defined by $SEM=\sigma \times \sqrt{1- Reliability}$ with *σ* the baseline standard deviation. The reliability is usually estimated using an internal consistency estimate, for example Cronbach’s alpha, but some authors also use a test-retest reliability estimate (such as an Intra-Class Correlation coefficient) or, more anecdotally, a split-half reliability measurement. SEM is assumed to be fairly sample-independent [19], which is its best advantage: a growing standard deviation is balanced by a higher reliability. Some authors like Wyrwich et al. consider one SEM as an approximation of the MCID [20, 21], based on the analysis of few studies comparing the value of one SEM with established standards for clinically relevant intra-individual change of HRQoL scores.

A related measure is the *Minimal Detectable Change (MDC)*, which gives a 95% confidence interval around the value of the score: ${MDC}_{95}=1.96\times \sqrt{2}\times SEM$. Some authors use the value of 1 MDC as an estimate of the MCID of a PRO instrument [22]. The MDC can be considered also as an SDD but can be useful to compare it with anchor-based estimates. Indeed, if a difference is larger than the MDC, it reflects a change larger than a difference that may occur due to an error of measurement with 95% confidence [13, 23, 24].

#### Anchor-based methods

The anchor-based approaches use an external indicator to define patient’s evolution. Usually, anchors are presented as Global Rating of Changes (GRC), a change measured by a single item, most of the time a Likert scale. The GRC is often completed by the patient himself, but sometimes by a relative or clinician(s) [25]. Patient is then assigned into several groups ranging from large negative to large positive changes in clinical or health status. Usually, it’s recommended to estimate different values of MCID for improvement and deterioration, since they are not symmetrical [26].

##### Correlation between the GRC and the observed change

A good GRC should be appropriately linked with the score difference. In the field of behavioral sciences, Cohen defined a correlation as small when it is between 0.10 and 0.30, medium between 0.30 and 0.50 and large above 0.50. Thus, the minimal correlation of 0.30 between the GRC and the measured score difference that is usually recommended in the literature [10] corresponds to a moderate correlation, initially proposed for behavioral science, and without any evidence but rule of thumb.

When the GRC is an ordinal variable, serial correlation coefficients should be computed to examine the association between anchor and score difference.

##### Estimation of MCID

We used different approaches, using two or three repeated measures of the PRO to estimate MCID.

1. *Two-measure approaches*

*Mean change score:* MCID was first estimated by the mean change score of patients who felt a little change (improvement or deterioration) at follow-up visit, with 95% confidence interval calculated on the base of its SD. This is the approach initially proposed by Jaeshcke et al. [2]. A closely-related approach was considered by Redelmeier et al.: the “mean change method” [27, 28]. Here, the MCID is the difference between the mean change score of patients who felt a little change (improvement or deterioration) at follow-up and the mean change score of patients who felt no change. The purpose is to adjust for possible bias in ratings. As shown below, the mean change score of patients who felt no change for our outcome of interest was 1 point. Thus, we did not report MCID estimates using Redelmeier estimate because it would have resulted only in a systematic translation of 1 point as compared to Jaeshcke approach.

If the aforementioned anchor-based estimates are based on averaging the distribution of the change using data from the subgroups of patients who felt little change, other anchor-based estimates were proposed and are commonly used [6, 8]. Usually these other estimates are trying to find a cut point discriminating best patients with little change from patients with no change.

*Receiver Operating Characteristic (ROC) curve:* We obtained ROC curve by comparing patients with little improvement versus those who were unchanged or worsened, or comparing patients with little deterioration versus those who were unchanged or improved to get the ability of the score difference to discriminate a patient as little changed in a direction (e.g., slightly improved) or unchanged/changed in the other direction (e.g., unchanged or worsened).

We estimated the Area Under the Curve (AUC), reflecting whether the score difference correctly distinguished patients who changed a little and those who didn’t (as classified by the anchor).

The best threshold was chosen to minimize classification errors, i.e. to find the best compromise between sensitivity and specificity. Youden index (the farthest point from the diagonal line) and the Closest point to the Top-Left (Euclidian distance) were used to determine the MCID. 95% confidence intervals of the cut-off point values was estimated using bootstrap.

*Intersection between two distribution curves:* In 2010, Gerlinger at al. proposed another discrimination technique: the cut point is defined as the intersection of the density curves of “little changed” (better or worse) patients and unchanged patients. In their paper, it was determined by non-parametric discriminant analysis [29]. If this method does not make any assumption about the shape of the distribution of the change in score in the subgroups, it was not detailed. We propose here a parametric equivalent. It makes an assumption on the shape of the distributions but it can be solved analytically with ease Assuming two normal distributions with means *μ*1 and *μ*2 and standard deviations *σ*1 and *σ*2, we can write the equation of their intersection: $\frac{1}{\sigma 1\times \sqrt{2\pi }}{e}^{-\frac{1}{2{\left(\frac{x-\mu 1}{\sigma 1}\right)}^2}}=\frac{1}{\sigma 2\times \sqrt{2\pi }}{e}^{-\frac{1}{2{\left(\frac{x-\mu 2}{\sigma 2}\right)}^2}}$.

After development, we obtain *ax*2 + *bx* + *c* = 0, with *a* = (*σ*1 − *σ*2); *b* = (2*μ*1. *σ*2^2^ − 2*μ*2. *σ*1^2^) and *c* = *σ*12. *μ*22 − *μ*12. *σ*22 − 2*σ*12. *σ*22 (*lnσ*1 − *lnσ*2).

Given the solution of an equation with two unknowns *∆* = *b*2 − 4*ac*, we can deduce the two points where the distribution curves cross: $x=\frac{-b\pm \surd \Delta  }{2a}$. These points represent the cutoff where the probabilities of having no change or either little improvement or worsening are equal.

**Fig. 1:**
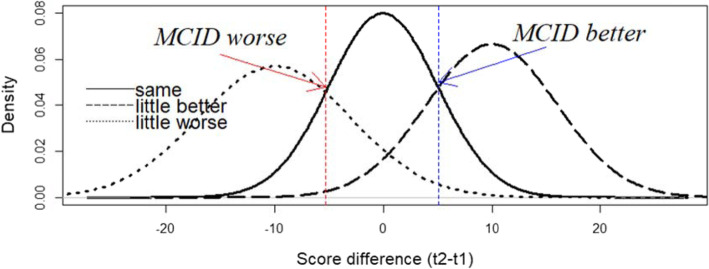
Distributions of the patients with no change, little improvement and little degradation between first (t1) and second measurement (t2): intersection points are here considered as a possible estimate for MCID

We estimated parametric distributions’ densities of the patients with no change/little improvement/little degradation, as we had the mean and the SD of each of these categories. We assumed that the score difference was following a normal distribution. An example is provided in the Fig. 1.

2. *Several (> 2) measures approach*

**Fig. 2:**
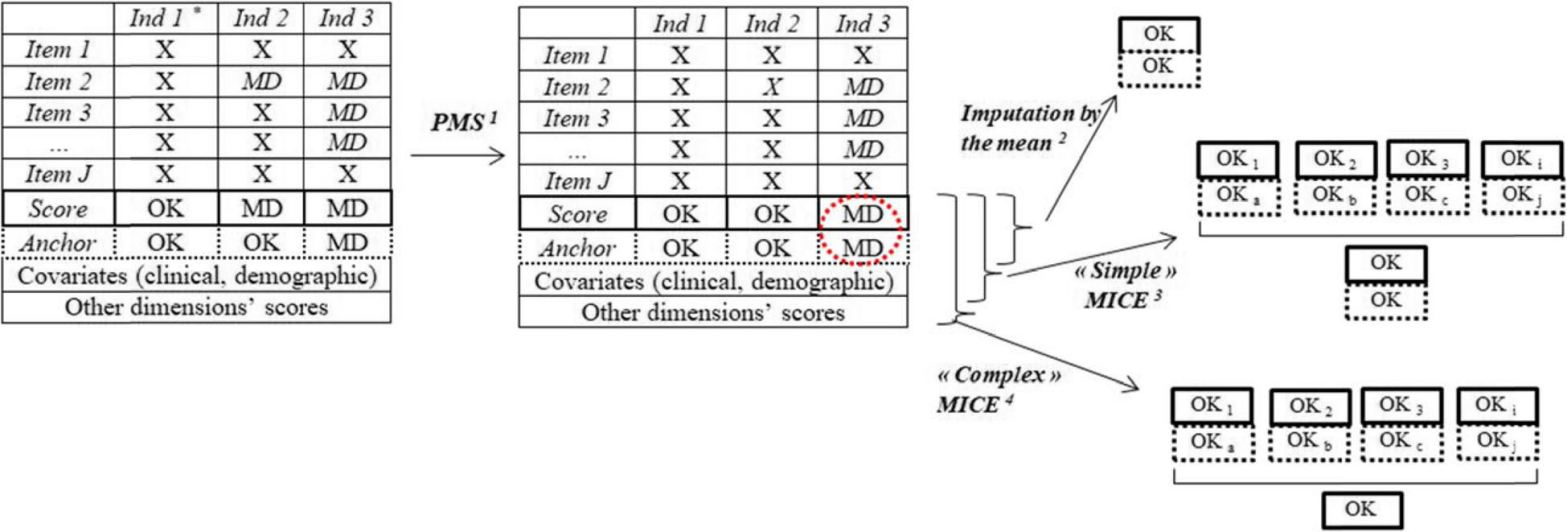
Illustration of the imputation methods used. *MD* Missing data. *X* completed item; *MICE* Multivariate Imputation with Chained Equations. ^1^*PMS* Personal Mean Score : each item is imputed ^2^Missing data in scores were imputed by the mean of observed scores ; missing data in anchor were imputed using a random sample weighted with observed probabilities of answers at corresponding anchor. ^3^Missing scores were imputed using personal mean matching, missing anchors were imputed using a polytomous regression, both using clinical and demographic variables. ^4^Missing scores were imputed using personal mean matching, missing anchors were imputed using a polytomous regression, both using clinical and demographic variables, and all scores from other dimensions

Very few studies about MCID estimation on empirical dataset are based on more than two times of measurement [8]. Most of the times they don’t take the correlation of repeated measures into account and simply estimate an overall mean pooling data of each time of measurement [8]. To our knowledge, we identified one study with repeated measures with a fully described and written appropriate model to deal with the multiple time points [30]. This approach can be advantageous, since it incorporates more data into MCID estimation, resulting in a potential improved precision if the correlation of repeated measures is properly accounted for.

Using repeated measures, we constructed a mixed linear regression, with a random effect on the individual (random intercept model) to estimate the mean change score in each category of patients (those who felt a little better, stable, or little worse), which was considered as the MCID (Eq. (1)):

1 $$ {\Delta }_{score\ i\left[j\right]}={\beta}_{anchor\ \left[j\right]}+{u}_i+{\epsilon}_{i\left[j\right]}\ \mathrm{with}\ {u}_i\sim N\left(0,B\right);{\epsilon}_i\sim N\left(0,{\sigma}^2\right). $$

With *∆*_*score i*[*j*]_ the score difference between two times of measurement for the *i* individual who would meet the *j* level of the GRC; *β*_*anchor* [*j*]_ the fixed effect parameter associated with the anchor at level *j*, *u*_*i*_ the coefficient associated with the random intercept effect on the individual, and *ϵ*_*i*_ the residual.

### Considering the baseline score

To determinate whether considering the baseline score was meaningful to estimate MCID, we also ran a mixed linear regression using the baseline score (cut into tertiles) as a fixed effect, and an interaction between baseline score and the GRC (Eq. (2)):

2 $$ {\Delta }_{score\ i\left[j,k\right]}={\beta}_{anchor\ \left[j\right]}+{\gamma}_{anchor\times score\_ bs\ \left[j,k\right]}+{u}_i+{\epsilon}_{i\left[j,k\right]}\ \mathrm{with}\ {u}_i\sim N\left(0,B\right);{\epsilon}_{i\left[j,k\right]}\sim N\left(0,{\sigma}^2\right). $$

With *∆*_*score i*[*j*, *k*]_ the score difference between two times of measurement for the *i* individual who would meet the *j* level of the GRC and would have a baseline score at a level *k*; *γ*_*anchor* × *score* _ *bs* [*j*, *k*]_ the interaction term between the baseline score at level *k* and the GRC at level *j*, and *ϵ*_*i*[*j*]_ the residual.

We used a likelihood-ratio test to compare Model (1) with Model (2) (i.e. without and with the interaction between baseline score and anchor as a fixed effect). We used a *p*-value < 0.05 as the threshold of statistical significance between the two likelihood values. If statistical significance was reached: it was used as an assessment of the need to consider baseline dependency of the MCID estimate. The MCID was estimated by linear combination of coefficients, with estimates of parametric 95% confidence interval.

### Missing data management (Fig. 2)

Numerous longitudinal studies with PRO data have missing data. When these data are used to estimate MCID, almost all the times, missing data are never dealt with and statistical analyses are conducted on the complete case sample [8]. Depending on the mechanism (i.e. missing data can be missing completely at random (MCAR), missing completely at random conditionally on observed variables (CD-MCAR), missing at random (MAR), or missing not at random (MNAR) according to Little and Rubin’s classification [31]), missing data will always have an impact on precision (due to loss of data) and can biased the results. Complete case analysis will always result in a loss precision but will not be biased only if missing data are MCAR. However, in the context of longitudinal PRO data collection, MCAR can be unlikely to assume.

First, we imputed missing items with Personal Mean Score (PMS), which consists in the imputation of missing items by the average of the items of the same dimension answered by the individual, if more than half of its items are filled [32].

Then, we described the demographic and clinical characteristics of patients with and without missing values at each visit. The typology of missing data was described (Online only supplement), as well as their potential mechanisms by comparing, at each visit, the variables between those who had and those who hadn’t missing data for the score (proportions were compared using Chi-square or Fisher test, and means were compared using Student T-test).

We first made our analyses only considering complete cases. Due to the nature of PRO, we expected a significant rate of missing data, so we planned to impute them using several imputation techniques to conduct sensitivity analyses. In the specific context of MCID estimation, imputation techniques require practical careful considerations, because not only quantitative scores have to be imputed, but also responses to the GRC which are on a Likert-scale.

First, we applied a simple imputation model, using the mean of observed scores to impute missing scores; the missing GRC responses were imputed using a random sample weighted with observed probabilities of answers. This simple imputation technique can prevent the loss of precision of a complete case analysis but assume MCAR mechanism. If the true mechanism generating missing data is not MCAR, MCID estimates are biased [33].

Second, missing scores and GRC responses were imputed using Multiple Imputation by Chained Equation (MICE). If MICE techniques are recommended for CD-MCAR or MAR missing data, they will not result in bias only if the models for imputations are adequately specified [34]. We explored different strategies of modeling imputations here. Missing scores were imputed using personal mean matching (to avoid imputed scores with out of range values), considering all clinical and demographic data and scores (from previous and/or following visit). Missing GRC responses were imputed using a polytomous regression, considering all clinical and demographic data, and the score difference between the current visit and the first. Then, we applied a more “complex” MICE method, adding all scores from other dimensions of the PRO multidimensional questionnaire as predictors for imputations. Finally, we reiterated those two MICE procedure using only available information (i.e. without imputation of missing data other than scores or anchors).

### Illustrative example

All the aforementioned methods of MCID determination have been applied on real datasets, to figure out the variability on the subsequent values.

We illustrated our purpose with the results from LIGALONGO study [11], which was a French multicenter randomized trial conducted from 2010 to 2013 designed to compare two types of intervention in the treatment of symptomatic hemorrhoidal disease. Three hundred ninety-three patients were submitted to a clinical evaluation, and filled auto-questionnaires the day before intervention, then at 3 months (visit 5) and 12 months (visit 7).

### Subjective concept of interest and questionnaire used to measure it

We aimed to estimate the MCID of HRQoL related concept.

The French MOS-SF36 (v2) [32, 35] is a generic HRQoL 36-items questionnaire divided into eight subscales addressing physical, mental and social health, and one item assessing the health transition (HT). For the present study, the analyses were performed on the five items of the General Health (GH) subscale, since it showed the best correlation with the GRC based on the available data. Each of these items was rated on an ordinal scale with five categories. The five responses of each patient were summed and the result was recalibrated to a score ranging from zero (worst perceived general health) to 100. By dividing the sample in three groups of approximately equal sizes, GH scores at baseline were considered as low when ranged from 0 to 65, medium from 66 to 82, or high from 83 to 100.

We used the Health Transition (HT) item as the patient-based GRC, at follow-up visits 5 (3 months) and 7 (1 year), which was worded as: “Compared to one year ago, how would you rate your health in general now?”. The patient could choose among five responses: “Much better”, “Somewhat better”, “About the same”, “Somewhat worse” and “Much worse”.

All statistical analyses were done using R Software (v3.3.2) [36], with packages mice [37] and pROC. The estimation of SF-36 scores for each individual of the dataset were done using the Stata Software 13 [38] (sf36fr package [39]). We rounded up the obtained MCID estimations to the nearest integer, as this is the way we usually interpret scores.

## Results

**Fig. 3:**
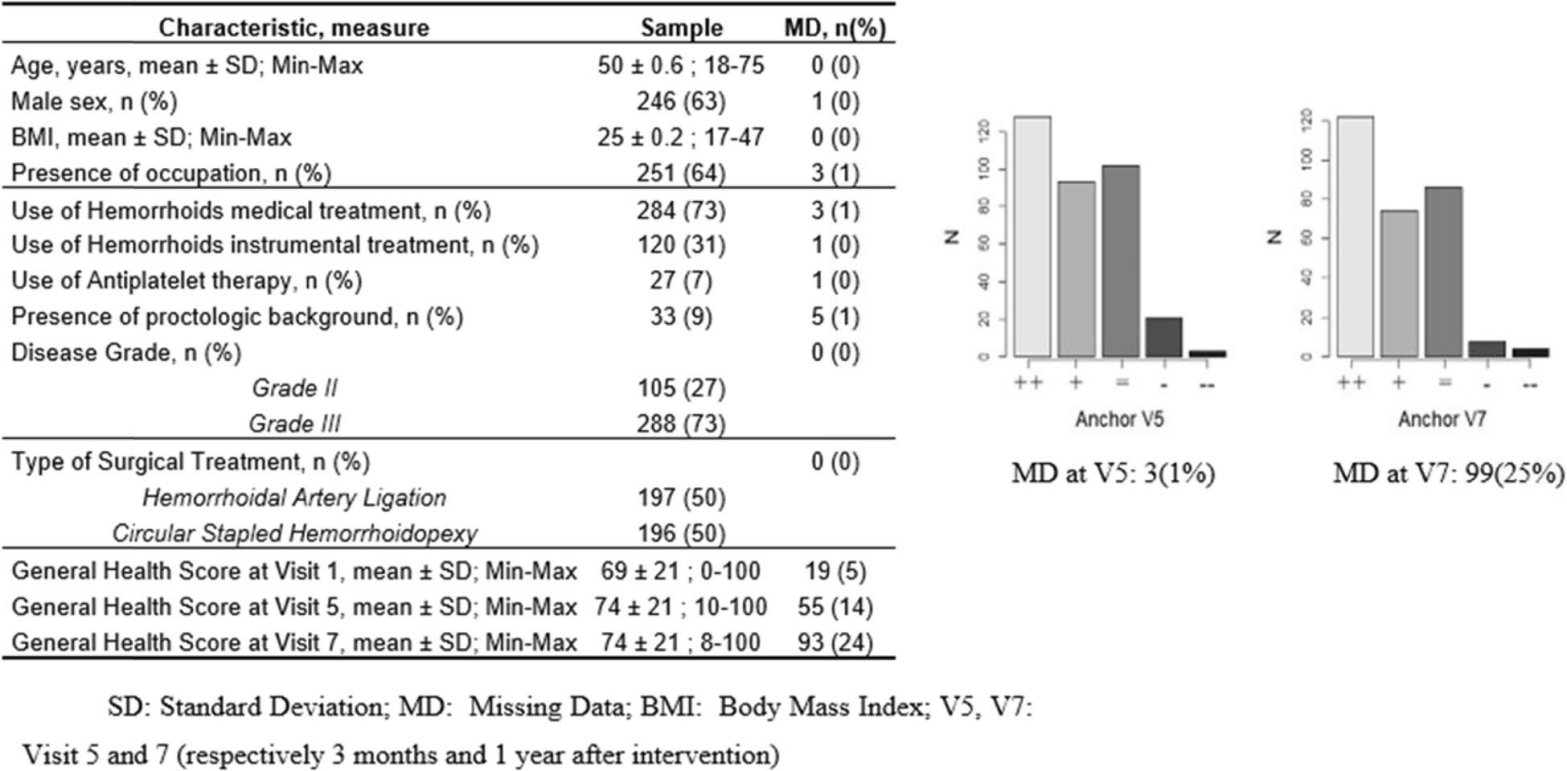
General characteristics of the LIGALONGO illustration study sample

Figure 3 shows the main characteristics, the mean GH scores and anchors at each visit of the Intention To Treat population of the LIGALONGO trial.

The GRC (HT) was adequately correlated with the GH score-difference at visit 5 and 7 (respectively, biserial correlation coefficient *r* = − 0.35 and − 0.32). The internal consistency of the GH score at visits 1, 5 and 7 was respectively *α* = 0.80, 0.85 and 0.83, which is consistent with the psychometric properties of SF-36 scale on French general population [32].

### Estimation of MCID: one and two measures- approaches

Table 1 shows the MCIDs obtained with different distribution-based methods.

**Table 1:** MCID estimations (with their 95% confidence interval) for different distribution-based methods applied to the LIGALONGO dataset using different imputation methods

	Distribution-based methods							
	0.5 SD_**b**_	1/3 SD_**b**_	0.2 SD_**b**_	SEM	MDC	0.5 SD_**ch**_	1/3 SD_**ch**_	0.2 SD_**ch**_
**Complete cases**	10 [10; 11]	7 [6; 7]	4 [4; 4]	9 [9; 10]	26 [24; 28]	8 [7; 9]	5 [4; 6]	3 [3; 4]
**Imputation by the mean**^**a**^	10 [9; 11]	7 [6; 7]	4 [4; 4]	9 [8; 10]	25 [23; 27]	8 [7; 9]	5 [5; 6]	3 [3; 4]
Simple MICE* imputation^**b**^	10 [10; 11]	7 [7; 7]	4 [4; 4]	9 [9; 10]	26 [25; 26]	8 [8; 8]	5 [5; 5]	3 [3; 3]
**Complex MICE imputation**^**c**^	11 [10; 11]	7 [7; 7]	4 [4; 4]	9 [9; 10]	26 [25; 27]	8 [8; 8]	6 [5; 6]	3 [3; 3]
**Simple MICE imputation (available information)**^**d**^	10 [10; 11]	7 [7; 7]	4 [4; 4]	9 [9; 10]	26 [25; 26]	8 [8; 8]	5 [5; 6]	3 [3; 3]
**Complex MICE imputation (available information)**^**d**^	10 [10; 11]	7 [7; 7]	4 [4; 4]	9 [9; 10]	26 [25; 26]	8 [8; 8]	5 [5; 6]	3 [3; 3]

Values in bracket are 95% Confidence Interval

*MICE* Multivariate Imputation with Chained Equations, *SD*_*b*_ Standard deviation at baseline score (visit 1), *SEM* Standard Error of Measurement, *MDC* Minimal Detectable Change, *SD*_*ch*_ Standard deviation of the difference score (score at Visit 5 – score at Visit 1)

^a^Missing scores were imputed by the mean-score, and missing anchors were imputed on the base of a weighted-probability

^b^Missing scores were imputed using personal mean matching, anchor was imputed using a polytomous regression, both using clinical and demographic variables, and GH scores

^c^Missing scores were imputed using personal mean matching, anchor was imputed using a polytomous regression, both using clinical and demographic variables, and all SF-36 scores

^d^The same MICE methods were applied, using only available information

There was a high variability between the different distribution-based methods, and the MCID of the GH-score was estimated to be between 3 and 26 points.

Table 2 shows different MCID values obtained with anchor-based methods. Considering the MCID as the mean GH score difference, it was estimated between 4 and 5 (95% CI ranging from 1 to 8 points) in the group who felt little improved, and between − 1 and 2 (95% CI ranging from − 10 to 5) in the group who felt little worsened.

**Table 2:** MCID estimated with different anchor-based methods and applied to the LIGALONGO dataset using different imputation methods

Method of imputation	MCID estimate	Groups (according to anchor at visit 5)											
		MCID Improvement					MCID Worsening					Unchanged	
		**MCID**	**95% CI**				**MCID**	**95% CI**				**Mean GH Δ**^**e**^	**95% CI**
**Complete cases**	**Mean**	4	[2; 7]				−4	[− 9; 2]				1	[−1; 3]
**Imputation by the mean**^**a**^		5	[1; 8]				−2	[−8; 3]				2	[−1; 4]
**Simple MICE imputation**^**b**^		4	[2; 7]				−4	[−9; 2]				1	[−1; 4]
**Complex MICE imputation**^**c**^		5	[2; 8]				−3	[−9; 2]				1	[−1; 4]
**Simple MICE imputation (available)**^**d**^		5	[3; 8]				−1	[−6; 5]				1	[−1; 3]
**Complex MICE imputation (available)**^**d**^		5	[2; 7]				−4	[−10; 1]				0	[−1; 3]
		**MCID**	**95% CI**				**MCID**	**95% CI**					
**Complete cases**	**Intersection of distribution curves**	8	[−7;15]				−3	[−10;12]					
**Imputation by the mean**^**a**^		14	[6;20]				−3	[−13;15]					
**Simple MICE imputation**^**b**^		9	[2;12]				−4	[−6;3]					
**Complex MICE imputation**^**c**^		8	[2;10]				−2	[−5;5]					
**Simple MICE imputation (available)**^**d**^		8	[4;10]				−10	[−24;12]					
**Complex MICE imputation (available)**^**d**^		7	[2;9]				−4	[−7;4]					
		**MCID**	**95% CI**	**AUC**	**Se**	**Sp**	**MCID**	**95% CI**	**AUC**	**Se**	**Sp**		
**Complete cases**	**ROC: closest from top-left corner**	5	[5; 5]	0.58	0.53	0.66	0	[−3; 5]	0.69	0.62	0.73		
**Imputation by the mean**^**a**^		5	[5; 5]	0.60	0.56	0.65	3	[−3; 5]	0.66	0.59	0.72		
**Simple MICE imputation**^**b**^		5	[2; 6]	0.61	0.57	0.64	2	[−3; 5]	0.69	0.61	0.72		
**Complex MICE imputation**^**c**^		5	[2; 7]	0.60	0.57	0.64	4	[−7; 16]	0.68	0.56	0.77		
**Simple MICE imputation (available)**^**d**^		5	[3; 7]	0.60	0.57	0.63	3	[−3; 6]	0.63	0.6	0.66		
**Complex MICE imputation (available)**^**d**^		5	[3; 7]	0.60	0.57	0.64	2	[−3; 5]	0.69	0.61	0.71		
		**MCID**	**95% CI**	**AUC**	**Se**	**Sp**	**MCID**	**95% CI**	**AUC**	**Se**	**Sp**		
**Complete cases**	**ROC: Youden**	5	[−3; 13]	0.58	0.48	0.72	0	[−5; 13]	0.69	0.60	0.76		
**Imputation by the mean**^**a**^		7	[5; 13]	0.60	0.51	0.72	3	[−3; 12]	0.66	0.57	0.76		
**Simple MICE imputation**^**b**^		6	[0; 11]	0.61	0.52	0.70	3	[−8; 13]	0.69	0.58	0.76		
**Complex MICE imputation**^**c**^		7	[1; 12]	0.60	0.52	0.70	2	[−3; 5]	0.68	0.60	0.72		
**Simple MICE imputation (available)**^**d**^		7	[−1; 12]	0.60	0.53	0.68	2	[−11; 14]	0.63	0.61	0.66		
**Complex MICE imputation (available)**^**d**^		6	[−1; 12]	0.60	0.53	0.69	3	[−7; 14]	0.69	0.57	0.77		

*MCID* Minimal Clinically Important Difference, *MICE* Multivariate Imputation by Chained Equations, *CI* Confidence Interval, *AUC* Area Under the Curve, *Se* Sensitivity, *Sp* Specificity, *ROC* Receiver Operating Curve

^a^Missing scores were imputed by the mean-score, and missing anchors were imputed on the base of a weighted-probability

^b^Missing scores were imputed using personal mean matching, anchor was imputed using a polytomous regression, both using clinical and demographic variables, and GH scores

^c^Missing scores were imputed using personal mean matching, anchor was imputed using a polytomous regression, both using clinical and demographic variables, and all SF-36 scores

^d^The same MICE methods were applied, using only available information

^e^Mean GH difference between visits 1 and 5 within the unchanged group of patients

Table 2 shows the MCID values estimated considering the intersection of distributions’ density curves for patients with no change/little improvement/little degradation (based on their answer to the GRC of the visit 5).

Considering MCID as the best threshold of a ROC curve (Table 2), we obtained different results whether we chose the closest threshold from the top-left or the Youden threshold. This was illustrated by the low AUC, which ranged between 0.58 and 0.61 for the “little better” group, and between 0.63 and 0.69 for the “little worse” group.

### Estimation of MCID using a three-measures approach

Table 3 reports the MCID for improvement and deterioration, for the whole sample, and within each groups of patients on baseline GH score (low, medium or high), using information from the three times of measurement. For improvement only, compared to the anchor-based mean approach with two times only (Table 2), it led to an increase in precision of the estimates (i.e. narrower CI), with estimates of a lower magnitude (1 to 4 points instead of 4 to 5).

**Table 3:** MCID estimated by the coefficients of linear-mixed effect model, considering baseline General Health scores, and applied to the LIGALONGO dataset using different imputation methods for missing data

	MCID for improvement				MCID for deterioration			
	All range baseline GH^**a**^	Low baseline GH^**b**^	Medium baseline GH^**b**^	High baseline GH^**b**^	All range baseline GH^**a**^	Low baseline GH^**b**^	Medium baseline GH^**b**^	High baseline GH^**b**^
**Complete cases**^**i**^	3 [1; 5]	12 [8; 15]	2 [−2; 5]	−7 [− 12; − 3]	− 10 [− 15; − 5]	− 4 [− 10; 3]	−16 [− 27; − 5]	− 17 [− 27; − 7]
**Imputation by the mean**^**ic**^	3 [1; 5]	14 [10; 18]	− 1 [− 7; 5]	−13 [− 19; − 6]	− 11 [− 16; − 6]	5 [− 3; 13]	− 8 [− 20; 4]	− 19 [− 32; − 5]
**Simple MICE imputation**^**d**^	3 [1; 5]	12 [8; 15]	2 [− 2; 5]	−7 [− 11; − 4]	−8 [− 13; − 4]	−2 [− 8; 5]	− 15 [− 24; − 5]	− 17 [− 26; − 7]
**Complex MICE imputation**^**e**^	3 [1; 6]	10 [7; 14]	1 [− 2; 4]	− 6 [− 10; − 3]	− 8 [− 13; − 4]	− 3 [− 8; 3]	−12 [− 18; − 7]	− 19 [− 25; − 13]
**Simple MICE imputation (available)**^**f**^	4 [1; 6]	13 [10; 16]	0 [− 3; 3]	− 6 [− 10; − 2]	−9 [− 14; − 4]	0 [− 5; 5]	−12 [− 18; − 7]	− 19 [− 24; − 13]
**Complex MICE imputation (available)**^**f**^	1 [− 1; 4]	10 [7; 13]	− 1 [− 4; 2]	−8 [− 11; − 4]	− 10 [− 15; − 5]	− 3 [− 8; 2]	−14 [− 19; − 8]	− 20 [− 26; − 15]

The presented scores were obtained with a linear-mixed effects regression, with a random effect on the individual (random intercept model) to estimate the mean change score in each category of patients (little better/ same/ little worse), and the baseline GH score as a fixed effect (+/− an interaction between baseline GH score and the anchor). The models including an interaction term are signaled with ^***i***^

*MICE* Multivariate Imputation by Chained Equations, *GH* General Health

^a^Minimal Clincally Important Difference estimated by the Mean GH-score difference between visits 1 and 5 or 1 and 7

^**b**^Minimal Clincally Important Difference estimated by the Mean GH-score difference between visits 1 and 5 or 1 and 7, according to each group of baseline GH score. Baseline GH scores are classified as low [0,65], medium (65,82] or high (82,100). The mean GH differences correspond to the fixed-effect associated coefficients of the patients who answered respectively “little better”, “little worse” and “same” at the anchor question. Values in bracket are Confidence Interval at a 95% level

^c^Missing scores were imputed by the mean-score, and missing anchors were imputed on the base of a weighted-probability

^d^Missing scores were imputed using personal mean matching, anchor was imputed using a polytomous regression, both using clinical and demographic variables, and GH scores

^e^Missing scores were imputed using personal mean matching, anchor was imputed using a polytomous regression, both using clinical and demographic variables, and all SF-36 scores

^f^The same MICE methods were applied, using only available information

In all dataset (complete or imputed ones), the Likelihood Ratio Test concluded to the dependence of the score difference on the baseline GH score (Table 3).

### Management of missing data

Comparison of demographic and clinical characteristics for patients with and without missing data at visits 5 and 7 are shown in eTables 1, 2, 3 and 4. We didn’t notice any difference in the two groups regarding all demographic characteristics, except for BMI at visit 5 (patients with missing data at visit 5 had a significantly higher BMI (=25.7 kg/m^2^) than those without missing data (=24.5 kg/m^2^)).

Concerning clinical characteristics, the only difference evidenced at visit 5 were for disease grade (patients with missing data had a lower grade disease (respectively, the ration of grade II/grade III disease was 40/60 in the missing data group, versus 24/76 in the complete-case group, *p* = 0.013). There also was a difference at visit 7 for disease grade (patients with missing data had a lower grade disease (respectively, the ration of grade II/grade III disease was 35/65 in the missing data group, versus 24/76 in the complete-case group, *p* = 0.029).

eTable 5 shows the amount of missing data at the different visits of the study. The amount of missing data for the difference in GH scores and GRC was substantial enough to justify the need to perform missing data imputation. In terms of mechanism, the observed relationships between the disease grade and missingness seems to imply a part of the missing data mechanism in this study is likely to be MNAR.

The imputed MCID estimates are displayed in Tables 1, 2 and 3. Imputation had no impact on MCID estimates with distribution-based methods. It had various impact for the other methods. For multiple imputation techniques, the greater variability was especially observed for the estimation of MCID for deterioration with intersection of distribution curve method, less with ROC (Table 2) and longitudinal models (Table 3). The simple imputation by the mean sometimes produced estimates very different from the others (intersection of distribution curve method (Table 1) or longitudinal models (Table 3)).

**Fig. 4:**
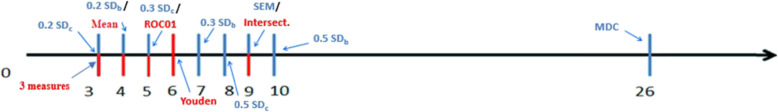
Variations of MCID values for improved patients (Missing data imputed by simple MICE). Note: Red are anchor methods. Blue are distribution-based methods. Missing scores were imputed using personal mean matching, anchor was imputed using a polytomous regression, both using a demographic nariables, and General Health scores. *SD*_c_ Standard Deviation of the change in scores. *SD*_b_ Standard of teh baseline score. *ROC01* Closet-point to rhe left of the reciever Operating curve diagram. *Intesect* Intersection point between the distributions of the change om scores between unchanged and improved patient. *SEM* Standard Error of Measurement. *MDC* Minimal Detectable Change

Finally, Fig. 4 summarizes the variability of MCID estimation using different methods (results displayed for improvement, with “simple” MICE imputation).

## Discussion

Illustrating our point by the LIGALONGO trial, we highlighted different issues in the MCID determination.

### The choice of method

Distribution-based methods don’t consider the patient’s feeling and generally are considered as worst compared to anchor-based methods [5]. Though, they offer some advantages; notably, SEM, MDC and estimations from baseline SD can be used to estimate MCID with a single time of measurement.

We can note in our example an agreement between techniques of MCID determination (Fig. 4 and Table 2). Hence, anchor methods for MCID for improvement stood around 4 and 7 points (except the intersection method), which corresponded to 0.2 to 1/3 SD_b_ or SD_c_. For distribution methods, MDC, SEM and 0.5 SD_b_ gave the higher MCID values.

As some authors do regarding MCID estimation [14], we suggest to first use anchor-based method, and to accompany it with a distribution-based method to enhance its accuracy (i.e a process sometimes called “triangulation”). Based on the agreement observed in this dataset, within distribution-based methods, we think that SEM, MDC and 0.5 SD_b_ or SD_c_ should be disregarded, in favor of 0.2 or 1/3 SD_b_ or SD_c_, since it returns values closer to those obtained with the most popular anchor-based methods (mean and ROC-curves [8]). This result is not in line with the agreement found by Wyrwich et al. [20, 21] between SEM estimate and anchor-based methods.

Considering ROC-based methods, we note in our dataset that MCID values for improvement were more precise but less discriminant for deterioration. Indeed, in LIGALONGO study, the expected evolution was a global improvement after the intervention for hemorrhoidal disease. Thus, patients with an improvement below their expectation would have been more likely to see themselves as unchanged. Hence, there was a thin difference between the thresholds of unchanged and improved patients, but the 95% CI was more accurate because there were a significant number of patients (*n* = 93 (26.8%)). Conversely, there was a clear difference between the thresholds of unchanged and slightly worsened patients, with a better AUC, but a large 95% CI, since there were few patients (*n* = 21 (6%)). Therefore, context (expected evolution in the sample used as data) is paramount to consider to correctly interpret MICD estimates.

The appropriate form of the function between change in score and responses to the GRC is unknown but could be complex. The now widely available machine learning techniques such as artificial neural network could be a potential tool to model this complexity. For example, those techniques could be used to train a classifier to predict if someone is a responder or not based on other available data rather than estimating a numerical MCID value and compared an individual change in score against this value. Nonetheless, such an approach would constitute a paradigm shift regarding the issue of the interpretation of a change in PRO score and would raise many unanswered questions [40].

### Management of missing data

We saw missing data imputation had different impacts, depending on which method for MCID determination was applied.

We chose to directly impute missing scores rather than missing items. Indeed, scores were already imputed by personal mean score if more than half of its items were filled (for example, three or more items for the GH dimension). Then, if scores were still missing, in fact more than half of its items were filled, and it represented a too big amount to impute with too few information.

We can note that imputation of missing data by the mean produced results that were out of step with other imputation techniques and complete cases. Hence, there were notable differences in estimated values with intersection points on density distributions, and in longitudinal models. These results can reflect the fact imputation by the mean is not appropriate when missing data are not MCAR, hence the current recommendations which advocate the use of multiple imputation techniques [31, 37]. Nonetheless, as noted above, we can hypothesize the mechanism at play in this study regarding missing data can, in part, be MNAR. Indeed, it was observed that patients with a lower disease grade tend to have missing data on PRO scores and GRC more often. Therefore, we can hypothesize some patients with a low disease severity did not come to follow-up visits because they did not feel the need of a close follow-up. To this date, even sophisticated imputation techniques such as MICE are biased when data are MNAR [33]. Thus, even after MICE imputation, we cannot rule out a slight (because we only found one relevant association between missingness and baseline characteristics) bias on MCID estimates. Therefore, there is a need to try to hypothesize what is the bias at stake here. If our assumption regarding disease severity is sound, it means missingness in this study was associated with a favorable outcome. Thus, we can expect missingness concerned patients who would have mainly answered having experienced a strong improvement. Therefore, for distribution-based anchor, we can hypothesize a slight underestimation of MCID estimates. For anchor-based estimates, it is trickier because statistical analyses are based on the data of subgroups: data from people who experienced large change are discarded. Thus, it is possible the aforementioned mechanism did not impact anchor-based estimates or impact it in the same way than distribution-based estimated.

### Correlation between the GRC and the score difference

The correlation between the HT GRC (“Compared to one year ago, how would you rate your health in general now?”) and the GH score difference was just above 0.30, which was correct, but maybe not optimal. This possibly explained the low values of AUC (which all were under 0.70). We hypothesized several reasons for this suboptimal correlation value.

The formulation of the GRC question influences its correlation with the score difference. The generic HT anchor was adapted to the measured concept (GH), since it verbalized the “health in general”. However, this GRC was not well-adapted for other concepts like physical functioning or mental health, which would have required specific wording to correctly measure it as a GRC.

The reference date was not clearly stated in the question HT, since it asked the patient “Compared to one year ago, how would you rate your health in general now?”. At one-year follow-up visit, it was unclear whether the patient should have compared his state with the period before or after the operation. Then, the reference date should be explicit on the GRC question. We can cite the Thyrqol study as an example (data not shown), which aimed at evaluating quality of life of patients, 2 and 6 months after their thyroidectomy. The GRC question explicitly stated the reference date as “before the operation”. Then, correlation coefficient between GH-score and HT GRC was 0.45 at 2 months, and 0.43 at 6 months visit. Even if it’s difficult to compare two studies with different populations, it appears that being precise in the formulation of the date could result in a better correlation between score difference and the GRC.

### The use of a three measures approach

For improvement, when using appropriate longitudinal mixed-level modeling to consider the available data from the three times of measurement, we observed an increase in precision (i.e. narrower CI) of MCID estimates. This result was plausible as one of the expected interests to use more than two-times of measurement is to base estimation on a higher quantity of data, therefore increasing precision. Nonetheless, compared to anchor-based mean on two-times of measurement only, there was also a change in the magnitude of MCID estimates. This variability can be the result of sampling hazard, but it could also be due to the reference date of the GRC question which always was “compared to one year ago” either at visit 5 or 7. Thus, responses to GRC with a different baseliner as reference could have been used by patients at the different visits, which can have led to bias in estimation. Therefore, if using data from multiple times of measurement with appropriate modeling can be an interesting way to enhance precision, the choice of the reference date for the GRC question must be dealt with caution: at each time of measurement: the GRC question should each time reference the same baseline.

We pointed the great dependence of the MCID on the baseline score, which is a quite-known phenomenon [17, 24, 26, 27].

### Limits of our study

We note that the mean GH difference in the group who felt no change was around 1 point and not 0. Although the 95% CI of this estimate contains zero, this could be a sign of response-shift [41, 42].

Like the Wyrwich et al. studies [20, 21] which suggested one SEM as an appropriate approximation of MCID values, our current study only bases its recommendations on the use of sample datasets whose population parameter values are unknown. Thus, the suggestions made here cannot be taken as experimental proofs of high epistemological values. Determining unbiased, or at least the Best Linear Unbiased Estimate of MCID should be approached by an experimental design with population parameters controlled by the researcher, such as Monte-Carlo simulation studies.

## Conclusion

As a conclusion, through the description of one study, we highlighted several issues in MCID determination. Currently, we recommend it should be estimated by an anchor-based method, accompanied by a distribution-based method, which should be 1/3 or 0.2 SD of either the baseline score or the score difference. To ensure a good correlation between anchor and the score difference, one should pay much attention to the formulation of anchor question and the reference date to which the patient is referred. Baseline score should be considered, since the score difference depends on it. In the case of several times (> 2) of measurement, it could be interesting to integrate all measures into a mixed-effects model. Finally, missing values, which are very often numerous in PRO-based studies, cannot be ignored. The choice of a method of imputation may directly influence the MCID values. Globally, we recommend using a MICE procedure for imputation, instead of imputation on the mean, but the modeling of the imputation procedure should be approached with great caution. MNAR mechanism in PRO longitudinal data can be frequently expected. Thus, a fair discussion of the potential bias on MCID estimates due to missingness should be engaged when appropriate. A high level of proof for the best MCID estimate is still needed. Monte-Carlo simulation studies can be an appropriate tool to help getting such a level of proof. Indeed, this type of experimental design allows a rigorous estimation of bias of many estimators against a “true” populational value that is controlled by the investigator. Moreover, the simulation of multiple scenarios can help investigating the variability of the statistical properties of estimators under various conditions (e.g. it can be a way of rigorously investigating the influence of the correlation between change in score and responses to the GRC on MCID estimation). Nonetheless, to perform such an experimental study, it would require first to formally define what is MCID as a statistical parameter with a definition in the population. It means a conceptual model is needed to describe what are the components engaged when someone has to answer to a PRO at multiple times of measurement and to a PGRC at the second time. From this model, a simulation model could be devised to simulate data with a known “true” MCID value. As part of the MIDIPRES project, these issues will be further investigated and future results of a such a simulation study will help in determining the appropriate way of estimating a MCID.

## Supplementary information

**Additional file 1: **Online only supplementary material. **eTable 1**. Comparison of patients with and without missing values on quantitative data at visit 5. **eTable 2**. Comparison of patients with and without missing values on qualitative data at visit 5. **eTable 3**. Comparison of patients with and without missing values on quantitative data at visit 7. **eTable 4**. Comparison of patients with and without missing values on qualitative data at visit 7. **eTable 5**. Availability of data at each visit and mechanism of loss of patients.

## Data Availability

Please contact author for data requests.

## Abbreviations

AUC: Area Under the Curve
BMI: Body Mass Index
CD-MCAR: Conditionally-Dependent Missing Completely At Random
CI: Confidence interval
ES: Effect size
GH: General Health
GRC: Global Rating of Change
HRQoL: Health-Related Quality of Life
HT: Health transition
MAR: Missing at random
MCAR: Missing completely at random
MCID: Minimal Clinically Important Difference
MDC: Minimal Detectable Change
MICE: Multiple Imputation by Chained Equations
MNAR: Missing not at random
MOS SF-36: MOS Short-Form 36 survey
PMS: Personal Mean Score
PRO: Patient-Reported Outcome
ROC: Receiver Operating Characteristic curve
SD: Standard Deviation
SD_b_: Standard Deviation of the baseline score
SD_ch_: Standard Deviation of the change in scores
SDD: Smallest Detectable Difference
SEM: Standard Error of Measurement
