# Supplementary material for: Practical issues encountered while determining Minimal Clinically Important Difference in Patient-Reported Outcomes
Source: Health Qual Life Outcomes. 2020 May 27;18:156. doi: 10.1186/s12955-020-01398-w (PMC7251729; doi:10.1186/s12955-020-01398-w)
Supplement: Supplementary file 1 — Additional file 1: Online only supplementary material. eTable 1. Comparison of patients with and without missing values on quantitative data at visit 5. eTable 2. Comparison of patients with and without missing values on qualitative data at visit 5. eTable 3. Comparison of patients with and without missing values on quantitative data at visit 7. eTable 4. Comparison of patients with and without missing values on qualitative data at visit 7. eTable 5. Availability of data at each visit and mechanism of loss of patients. [file 12955_2020_1398_MOESM1_ESM.docx]

**ONLINE ONLY SUPPLEMENTARY MATERIAL**

eTable 1. Comparison of patients with and without missing values on quantitative data at visit 5

|  | **Complete answers at Visit 5 (n=328)** | | **Incomplete answers at Visit 5 (n=65)** | |  |
| --- | --- | --- | --- | --- | --- |
|  | **Mean** | **SD** | **Mean** | **SD** | **p** |
| **Age (years)** | 50.1 | 12.0 | 51.3 | 12.8 | 0.46 |
| **Body Mass Index (kg/m²)** | 24.5 | 4.1 | 25.7 | 3.7 | 0.02 |
| **Pain at Visit 1** | 4.0 | 2.5 | 4.6 | 2.7 | 0.15 |
| **Disease score at Visit 1** | 2.4 | 0.82 | 2.5 | 0.8 | 0.58 |

eTable 2. Comparison of patients with and without missing values on qualitative data at visit 5

|  |  | **Complete answers at Visit 5 (n=328)** | **Incomplete answers at Visit 5 (n=65)** |  |
| --- | --- | --- | --- | --- |
|  |  | **N (%)** | **N (%)** | **p** |
| **Sex** | **Male** | 203 (62) | 43 (67) | 0.48 |
|  | **Female** | 125 (38) | 21 (33) |  |
| **Occupation** | **No** | 112 (34) | 27 (42) | 0.32 |
|  | **Yes** | 213 (66) | 38 (58) |  |
| **Haemorrhoids** | **No** | 84 (26) | 22 (35) | 0.12 |
| **medical treatment** | **Yes** | 244 (74) | 40 (65) |  |
| **Haemorrhoids** | **No** | 224 (68) | 48 (75) | 0.30 |
| **instrumental treatment** | **Yes** | 104 (32) | 16 (25) |  |
| **Antiplatetel therapy** | **No** | 309 (94) | 56 (88) | 0.06 |
|  | **Yes** | 19 (6) | 8 (12) |  |
| **Proctologic background** | **No** | 298 (92) | 57 (90) | 0.80 |
|  | **Yes** | 27 (8) | 6 (10) |  |
| **Disease Grade** | **Grade II** | 79 (24) | 26 (40) | 0.01 |
|  | **Grade III** | 249 (76) | 39 (60) |  |
| **Surgical treatment** | **HAL** | 162 (49) | 35 (54) | 0.59 |
|  | **SH** | 166 (51) | 30 (46) |  |

eTable 3. Comparison of patients with and without missing values on quantitative data at visit 7

|  | **Complete answers at Visit 7 (n=328)** | | **Incomplete answers at Visit 7 (n=65)** | |  |
| --- | --- | --- | --- | --- | --- |
|  | **Mean** | **SD** | **Mean** | **SD** | **p** |
| **Age (years)** | 50.8 | 12.0 | 49.0 | 12.4 | 0.21 |
| **BMI (kg/m²)** | 24.6 | 3.9 | 24.9 | 4.3 | 0.45 |
| **Pain at Visit 1** | 3.9 | 2.6 | 4.5 | 2.4 | 0.03 |
| **Disease score at Visit 1** | 2.4 | 0.9 | 2.5 | 0.7 | 0.70 |

eTable 4. Comparison of patients with and without missing values on qualitative data at visit 7

|  |  | **Complete answers at Visit 7 (n=328)** | **Incomplete answers at Visit 7 (n=65)** |  |
| --- | --- | --- | --- | --- |
|  |  | **N (%)** | **N (%)** | **p** |
| **Sex** | **Male** | 174 (61) | 72 (66) | 0.40 |
|  | **Female** | 109 (38) | 37 (34) |  |
| **Occupation** | **No** | 99 (35) | 40 (37) | 0.79 |
|  | **Yes** | 182 (65) | 69 (63) |  |
| **Haemorrhoids** | **No** | 75 (27) | 31 (29) | 0.62 |
| **medical treatment** | **Yes** | 208 (73) | 76 (71) |  |
| **Haemorrhoids** | **No** | 192 (68) | 80 (73) | 0.28 |
| **instrumental treatment** | **Yes** | 91 (32) | 29 (27) |  |
| **Antiplatetel therapy** | **No** | 261 (92) | 104 (95) | 0.26 |
|  | **Yes** | 22 (8) | 5 (5) |  |
| **Proctologic background** | **No** | 260 (92) | 95 (90) | 0.42 |
|  | **Yes** | 22 (8) | 11 (10) |  |
| **Disease Grade** | **Grade II** | 67 (24) | 38 (35) | 0.03 |
|  | **Grade III** | 216 (76) | 72 (65) |  |
| **Surgical treatment** | **HAL** | 142 (50) | 55 (50) | 0.97 |
|  | **SH** | 141 (50) | 55 (50) |  |

eTable 5. Availability of data at each visit and mechanism of loss of patients

|  | **GH Score V1** | **GH Score V5** | **GH Score V7** | **V5 - V1 GH**  **score difference** | **Anchor at V5** | **Anchor at V7** |
| --- | --- | --- | --- | --- | --- | --- |
| **Whole sample (n=393)** | 374 (95) | 338 (86) | 291 (74) | 328 (83) | 347 (88) | 294 (75) |
| **Patients with only V1 (n=22)** | 18 (82) |  |  |  |  |  |
| **Patients with only V1 and V5 (n=42)** | 41 (98) | 34 (81) |  | 33 (79) | 35 (83) |  |
| **Patients with only V1 and V7 (n=5)** | 4 (80) |  | 2 (40) |  |  | 2 (40) |
| **Patient available at all visits (n=324)** | 311 (96) | 304 (94) | 289 (89) | 295 (91) | 312 (96) | 292 (90) |

Note: Values are frequencies of available data for each variable. Values in brackets are percentage of available data for each variable, the denominator is the sample size of the corresponding line. GH = General Health. V1, V5 and V7 = Visit 1, 5 and 7.
